# Radar quantifies migrant concentration and Dawn reorientation at a Great Lakes shoreline

**DOI:** 10.1186/s40462-018-0135-3

**Published:** 2018-08-29

**Authors:** Kevin W. Heist, Tim S. Bowden, Jake Ferguson, Nathan A. Rathbun, Erik C. Olson, Daniel C. Nolfi, Rebecca Horton, Jeffrey C. Gosse, Douglas H. Johnson, Michael T. Wells

**Affiliations:** 1 U.S. Fish and Wildlife Service, Ecological Services, 5600 American Blvd. West, Ste. 990, Bloomington, MN 55437 USA; 2 Bureau of Land Management, Surprise Field Station, 602 Cressler St., Cedarville, CA 96104 USA; 3 Department of Fisheries, Wildlife and Conservation Biology, University of Minnesota, 135 Skok Hall, 2003 Upper Buford Circle, St. Paul, MN 55108 USA; 4 U.S. Fish and Wildlife Service, Eastern Idaho Field Office, 4425 Burley Drive, Suite A, Chubbuck, ID 83202 USA; 5 Minnesota Department of Natural Resources, Ecological and Water Resources, 1200 Warner Road, St. Paul, MN 55404 USA

**Keywords:** Migration, Radar, Aeroecology, Ecological barrier, Coastal ecology, Great Lakes

## Abstract

**Background:**

Millions of flying migrants encounter the Great Lakes and other large water bodies on long-distance flights each spring and fall, but quantitative data regarding how they traverse these obstacles are limited. Shorelines are known areas of migrant concentration due to the ecological barrier effect, but details on the magnitude of this concentration and the flight behaviors causing it are largely unknown and difficult to quantify. Mobile avian radar can provide a unique view of how birds and bats move across landscapes by tracking thousands of individual migrants moving through a sample volume that extends multiple kilometers in radius.

**Results:**

During the spring of 2014 we used two avian radar units to compare migration patterns at shoreline (1.5 km from the shore) and inland (20 km from the shore) sites along the eastern shoreline of Lake Michigan in the north-central US. We found shoreline activity to be 27% greater than inland activity over all time periods, and 132% greater during the hour surrounding dawn. An analysis of flight directions found that migrants flew to the north and northwest during dusk and night, with many heading out over the lake, but shifted direction towards the east at dawn, as those flying over water reoriented towards land. This shift in direction, which was most intense at the shoreline, may contribute to the higher concentrations of migrants observed at shorelines in this study and others.

**Conclusions:**

These findings help confirm and quantify the phenomenon of nocturnal migrant reorientation at dawn, and also stress the functional importance of coastal regions for aerial migrants. The high use of coasts by migrants highlights the importance of conserving shoreline stopover habitat, which often competes with anthropogenic uses. We suggest using a high degree of caution when assessing potential impacts from development in these sensitive environments, and encourage protection of these high-use areas.

**Electronic supplementary material:**

The online version of this article (10.1186/s40462-018-0135-3) contains supplementary material, which is available to authorized users.

## Background

Many species of birds and bats experience strong selective pressure during migration [1–3] and the need for conservation during this vulnerable life cycle phase is clear [4–7]. Identifying migration routes, habitats used, and causes of stress or mortality during this phase is as important as understanding the requirements of quality breeding and wintering grounds [8]. Nonetheless, much remains unknown about how migrating birds and bats connect distant habitats.

Technological advances and improved modeling techniques are beginning to close this information gap by identifying continent-level movement patterns as well as areas where migrants concentrate [9]. For example, NEXRAD radar and citizen science data suggest three broad flyways that are shaped by synoptic weather patterns and funnel migrants through regions of the continental United States [10, 11]. These movements are thought to occur in broad waves across the landscape, but not all habitats traversed are equally important. Within general flyways, migrant activity is concentrated along physiographic features such as rivers, ridgelines, or shorelines that are thought to be used for navigation or refuge [12–14]. Our ability to identify and protect a network of stopover habitats along these features is an important component of conserving migratory species.

Migrants within both the central and eastern flyways move through the Great Lakes region, and there is evidence that Great Lakes shorelines support a relatively high concentration of migrating birds and bats [13–16]. Migrants can concentrate along shorelines for a variety of reasons. For terrestrial birds and bats, the Great Lakes represent an ecological barrier: an area of inhospitable habitat separating suitable habitat [17] that a large portion of migrants must contend with during their long-distance journeys. Depending on environmental and physiological conditions, migrants might risk crossing a water body that impedes their progress, or circumnavigate it along the shoreline [14, 18, 19]. Additionally, shorelines or other leading landmarks that generally align with the direction of travel may assist orientation or navigation [13, 20]. Flight behaviors of nocturnal migrants may further depend on the time of night, with movement patterns during dusk (embarking on flights) and dawn (concluding flights) differing from those at night. Avoidance of water crossings during any period can result in the increased use of near-shore habitats, making shorelines important stopover areas. Shoreline habitats may provide staging or fall-out areas for migrants, which can be critical for successful migration [13, 21], and areas in the immediate vicinity of the coast can experience particularly high use [22]. In addition to providing refuge, lake shorelines also offer increased foraging opportunities relative to inland areas due to an abundance of emergent aquatic insects [23].

Radar has been used to observe nocturnal migrant reorientation towards shorelines along oceans [24, 25], seas [26], and along inland water bodies including the Great Lakes [14, 27]. Several recent studies in North America have examined migration patterns using a network of large-scale weather surveillance radars (WSR-88D) capable of measuring target density and movement over broad regions [13, 27–29]. The smaller-scale mobile radars used in this study provided a less expansive view of the airspace but offered distinct advantages over weather radar. Mobile radar units can be positioned in almost any location desired to survey particular landscape features or phenomena of interest. Additionally, our units were designed specifically to detect bird- and bat-sized targets rather than weather patterns. This resulted in the ability to count and track individual targets, up to several thousand simultaneously, providing a far more detailed description and quantification of migratory flight patterns.

Our objectives were to test the hypotheses that 1) migrant passage rates are greater along a Great Lakes shoreline than at inland sites at similar latitude, and that 2) changes in flight direction occurring at the shoreline contribute to observed differences in passage rates. We used count data from a vertical antenna in a regression slope analysis to test the first hypothesis, and target headings from a horizontal antenna in an analysis of flight directions to test the second hypothesis. The results of this research offer a more detailed view of the interplay between broad-front and corridor-based migratory movement. They provide a quantification of the effects of physiographic features in concentrating migrant activity. These findings are useful in informing conservation strategies along Great Lake shorelines as well as other coastal areas.

## Methods

### Study sites

**Fig. 1:**
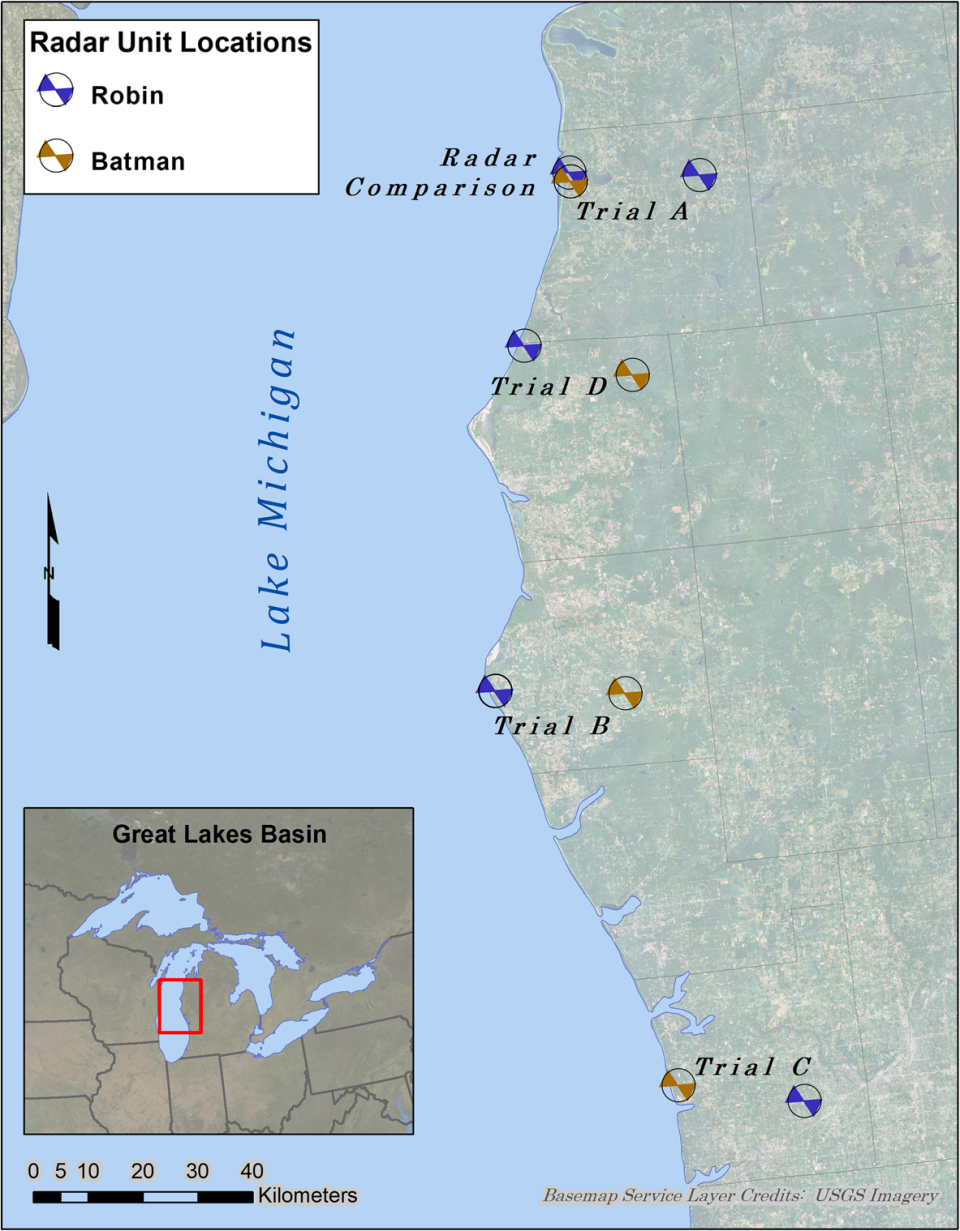
Radar locations on Lake Michigan for the spring 2014 migration season. Radar units Batman and Robin alternated between shoreline and inland for successive trials. Colored triangles depict the approximate orientation of the vertical antenna, which was 300° (north of west) at each site

During the spring of 2014 we placed two radar units along the eastern shoreline of Lake Michigan in the north-central US from Manistee to Ottawa counties, Michigan (Fig. 1). First, we conducted a radar comparison (RC) to estimate any differences in detection rate, the rate at which each unit detects and tracks targets, between the radar units by placing both units in close proximity along the shoreline in Manistee County, 1.5 km from each other and both 1.5 km from Lake Michigan. The purpose of the radar comparison was to test whether the radar units performed similarly enough to allow direct comparison of shoreline and inland observations without adjusting results for detection bias. After the radar comparison, the units were moved to paired locations, comprising one “shoreline” location 1.5 km from the lake and one “inland” location 20 km from the lake, at similar latitudes. A total of four trial periods (A, B, C, and D) with paired locations were sampled throughout the spring and into early summer. Radar units alternated between the shoreline and inland location on successive trials to further reduce any effect of differing detection rates. Trial locations were distributed along the coast with north-south separation of about 55 km between each pair of sites.

Selection of radar monitoring sites involved preliminary geographic analysis to locate areas with topography and land cover characteristics that would provide unobstructed radar views, and appropriate distances to the shoreline (1.5 km and 20 km). This was followed by on-site assessments to determine suitability for radar deployment and operation (see [30] for more detail on site selection and setup). Nine sites were selected for use during the initial radar comparison and four subsequent inland/shoreline comparison trials (Table 1). We used site 2 for both the radar comparison and trial A.

**Table 1:** Study Sites

Site	Trial	Type	Lat, Long	Dates	Radar Unit	Open Water
1	RC	Shoreline	−86.229, 44.449	April 16–April 22	Robin	23.0%
2	RC, A	Shoreline	−86.227, 44.434	April 16–May 8	Batman	21.2%
3	A	Inland	−85.932, 44.422	April 23–May 8	Robin	0.3%
4	B	Shoreline	−86.521, 43.614	May 9–May 21	Robin	30.1%
5		Inland	−86.229, 43.588		Batman	0.1%
6	C	Shoreline	−86.205, 42.937	May 22–June 1	Batman	31.3%
7		Inland	−85.927, 42.889		Robin	0.5%
8	D	Shoreline	−86.373, 44.174	June 2–June 23	Robin	29.3%
9		Inland	−86.134, 44.107		Batman	0.3%

Nine sites used for a radar comparison (RC) and four subsequent trial periods (A, B, C, and D) along eastern Lake Michigan during spring 2014. Shoreline sites were 1.5 km inland and Inland sites were 20 km inland. Percent open water indicates amount of open water within a 3.7 km radius (approximate maximum radar detection range), according to the 2011 National Land Cover Database [59]

### Data collection

**Fig. 2:**
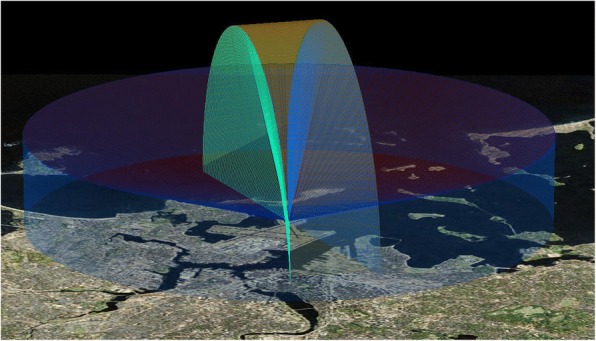
Computer representation of the survey volume scanned by horizontal and vertical radars. Horizontal antenna coverage (blue) reached approximately 3.7 km from the radar and the vertical antenna sweep (green) reached approximately 2.8 km up into the sky. Graphic provided by DeTect, Inc.

We used two model SS200DE MERLIN Avian Radar Systems (DeTect Inc., Panama City, FL) named “Batman” and “Robin” to observe migration movements. These systems were selected because they are self-contained mobile units specifically designed to detect, track, and count individual bird and bat targets. The systems use S-band frequencies, which are less sensitive to contamination from insects and precipitation than other bands used for radar ornithology [31]. Each radar unit employed two marine radar antennas that operated simultaneously: a horizontal surveillance radar (HSR) that scanned the horizontal plane from 0 to approximately 20° above the horizon, and a vertical scanning radar (VSR) that scanned a slice of the sky 26° wide (Fig. 2). Landscape features and vegetation surrounding the radar block radar signals emanating from the antennas at low angles (near the horizon), resulting in low detection rates for targets flying below 100 m above ground level on both HSR and VSR. The HSR had a maximum detection range of approximately 3.7 km, and the VSR had a maximum range of about 2.8 km. Detection probability varies with distance from the radar as well as target size, so these maximum ranges likely only apply to large birds or flocks of small birds.

We deployed the radar units for a side-by-side radar comparison (RC) for one week in mid-April to test for any differences in detection rates. Trials comparing shoreline and inland activity began immediately following the radar comparison. Data collection continued until late June, 2014. Each trial lasted about two weeks to allow enough time for several nights of high-migration activity (Table 1). Trial D lasted an additional two weeks into late June to ensure that the end of migration season had been covered. Biologists visited the site regularly during data collection to ensure continuous function, monitor raw and processed radar outputs, conduct routine maintenance, and manage data storage.

The VSR antennas were oriented with similar azimuth at each site and trial, with VSR sample volumes extending approximately east-southeast (120°) to west-northwest (300°) at each site. This orientation was selected to optimize detection of targets flying in the anticipated northward direction for spring migration, while also reducing double-counting which occurs when a single bird or bat is counted as a unique record two or more times as it passes through the sample volume. Each antenna completed a scan of the sample volume every three seconds. Returns from stationary objects within the sample volume (clutter) were removed via a clutter-mapping procedure executed prior to data collection at each site. MERLIN software (DeTect Inc., Panama City, FL) analyzed sequences of dynamic radar returns for each antenna separately, recording measurements of size, shape, location, speed, and heading of potential targets. Tracking algorithms assessed whether a sequence of returns fit the movement characteristics of a biological “target” such as a bird or bat. If identified as a target, the software connected the sequence of returns to form a track through the sample volume indicating its flight path. To reduce false positives, the software removed tracks with fewer than five sequential observations. The system was designed and calibrated to detect birds (including small passerines) and bats, but does not differentiate between the two or provide any taxonomic classification.

Outputs from the tracking software included visualizations depicting target tracks over a specified period of time (Trackplots) for VSR and HSR, and a database containing characteristics of targets such as position (for both VSR and HSR), flight direction and velocity (for HSR). Visual review of 15-min Trackplots facilitated examination of general characteristics of movement and migration intensity, as well as specific flight patterns related to physiographic features or timeframes. Trackplots were also used to identify periods of rain or contamination from non-biological targets, and those periods were removed from the dataset prior to analysis. The database provided target counts, directions, and summary statistics used for quantitative analyses.

### Data analysis

Data from each antenna were used for different analyses. VSR counts were used as an index of activity because VSR is less subject to double-counting targets due to the shape of its sample volume (Additional file 1). Only targets detected within a “standard front” extending 500 m to each side of the radar unit were included in the analysis of VSR data. This type of metric has been used in radar ornithology as a means to standardize comparisons among studies [32–34]. HSR data are not a reliable indicator of activity, but do provide the flight direction of each target. For an analysis of flight directions, we examined the circular distribution of HSR targets rather than the quantity of HSR targets. For that reason, directional analyses were carried out with equal-sized random samples of HSR targets. Samples were taken from the entire HSR sample volume.

To examine circadian patterns, we defined four biological periods: we defined “dawn” as 30 min before sunrise to 30 min after sunrise, “day” as 30 min after sunrise to 30 min before sunset, “dusk” as 30 min before sunset to 30 min after sunset, and “night” as 30 min after sunset to 30 min before to sunrise. Dusk and dawn periods were always one hour long, but day and night varied in length. Day was 12 h, 52 min at the beginning of trial A, and 14 h, 32 min at the end of trial D. Night was 9 h, 8 min at the beginning of trial A and 7 h, 28 min at the end of trial D.

Our analysis focused primarily on time periods of peak migration, which occurred at night. Other biological periods were used to compare activity levels relative to nocturnal migration and changes in flight behavior at the beginning and end of nocturnal flights. In particular, we were interested in examining how the lake’s ecological barrier effect might influence passage rates or flight direction during dusk and dawn when compared to nighttime activity.

#### Radar comparison and migration activity

We used target passage rate (TPR) as an index of flight activity from VSR data. TPR was calculated as the average number of targets passing through a 1 km-wide vertical standard front in one hour, and is comparable to migration traffic rate (MTR) used in other studies. For dawn and dusk, TPR is equal to the target count for that hour. For day and night, it is the mean hourly passage rate over the time period. Analysis of data from both the radar comparison and the shoreline-inland trials was carried out with regression slope tests (Additional file 2) in program R [35].

For the radar comparison, hourly TPR from Batman (y) were regressed on hourly TPR from Robin (x) to test for differences in detection rates. For the trials, shoreline TPR (y) were regressed on inland TPR (x) within each biological period to assess differences in activity levels. If there is no difference between x and y, the slope of the regression line will equal 1, with y-intercept at 0. However, if the regression line differs to the extent that its 95% confidence region does not include the line y = x, then there is evidence of a significant difference between inland and shoreline activity. Because the TPR values used in each analysis are effectively two interdependent measurements of one larger phenomenon (migration intensity), with no clear or presumed causal relationship between the two, designation of predictor and response variables was arbitrary. For this reason, we used least rectangles regression to incorporate errors from both variables into regression line fitting [36]. Exploratory analysis revealed TPR to have a right-skewed distribution common to count and rate data, so Poisson least rectangles regression was used to estimate the regression line parameters [37]. To reduce reliance on parametric assumptions about the error distribution associated with our regression estimates, a non-parametric bootstrap method was used to estimate the confidence intervals for slope and intercept [38, 39], producing a 95% confidence region around the regression line.

#### Direction of flight

Differences in migration intensity across the landscape may result from migrants altering their flight paths in response to geographic features, leading to concentration along barriers or obstacles. We used data from the HSR to assess the general directionality of flight during the night and examine whether flight directions changed in the early morning hours, as nocturnal migrants over water returned to the shoreline to land (Additional files 3 and 4). To test the hypothesis that migrants turn toward shore at dawn, we tested for differences in mean direction of flight between night and dawn, at both inland and shoreline sites. A turn towards shore would be indicated by a more easterly direction of flight at dawn, relative to northerly flight at night. We expected the change to be more intense at the shoreline if it were caused by avoidance of open water. Because the shoreline radar sites had sample volumes that were only partially over water, we also analyzed a subset of the shoreline data that included only targets traveling over open water. “Over water” targets were identified by their longitude relative to the north-south shorelines at our study sites. Over-water targets were defined as those tracked west of longitude − 86.249° at site 2 (trial A), west of longitude − 86.538° at site 4 (trial B), and west of longitude − 86.218° at site 6 (trial C). Data from trial D were excluded from analysis prior to site comparisons (see Results).

The radar tracking software assigned each target a direction from 0 and 359 degrees, with 0 representing north and ascending clockwise. Horizontal radar data are subject to high rates of double-counting due to the shape of the sample volume and effects of ground clutter, which creates radar blind-spots. As a result, target counts were not used for direction analysis. To compare the directional distributions of HSR targets, equal-sized random samples of 20,000 targets from each of two time periods (night and dawn), three locations (inland, shoreline, and over water) and three trials (A, B, and C) were used for statistical tests (*n* = 360,000). This sample size was chosen in order to include a large number of targets in each sub-group for accurate representation of directional variation, while allowing for the same number of targets to be sampled from groups of different sizes, the smallest of which was just over 20,000. We used circular statistics in R package ‘circular’ [40], specifically Watson-Williams tests, to test for differences in mean direction of flight between night and dawn. Due to large sample sizes, small differences in means were expected to produce statistically significant results, so we considered *p*-values less important than the magnitude of shifts in direction as an indicator of biological significance.

## Results

The two radar units collected 2643 h of data at nine study sites over the 70-day season. Flight traffic was most intense during the night and dawn, corresponding with the peak and end of nocturnal migration pulses. Even though night accounted for only a third of the total study period due to long springtime days, 85.8% of all targets were detected during the night, averaging 545 targets per hour. Dawn, day, and dusk accounted for 1.73, 11.7, and 0.74% of targets, averaging 112, 38, and 35 targets per hour respectively.

### Radar comparison

**Fig. 3:**
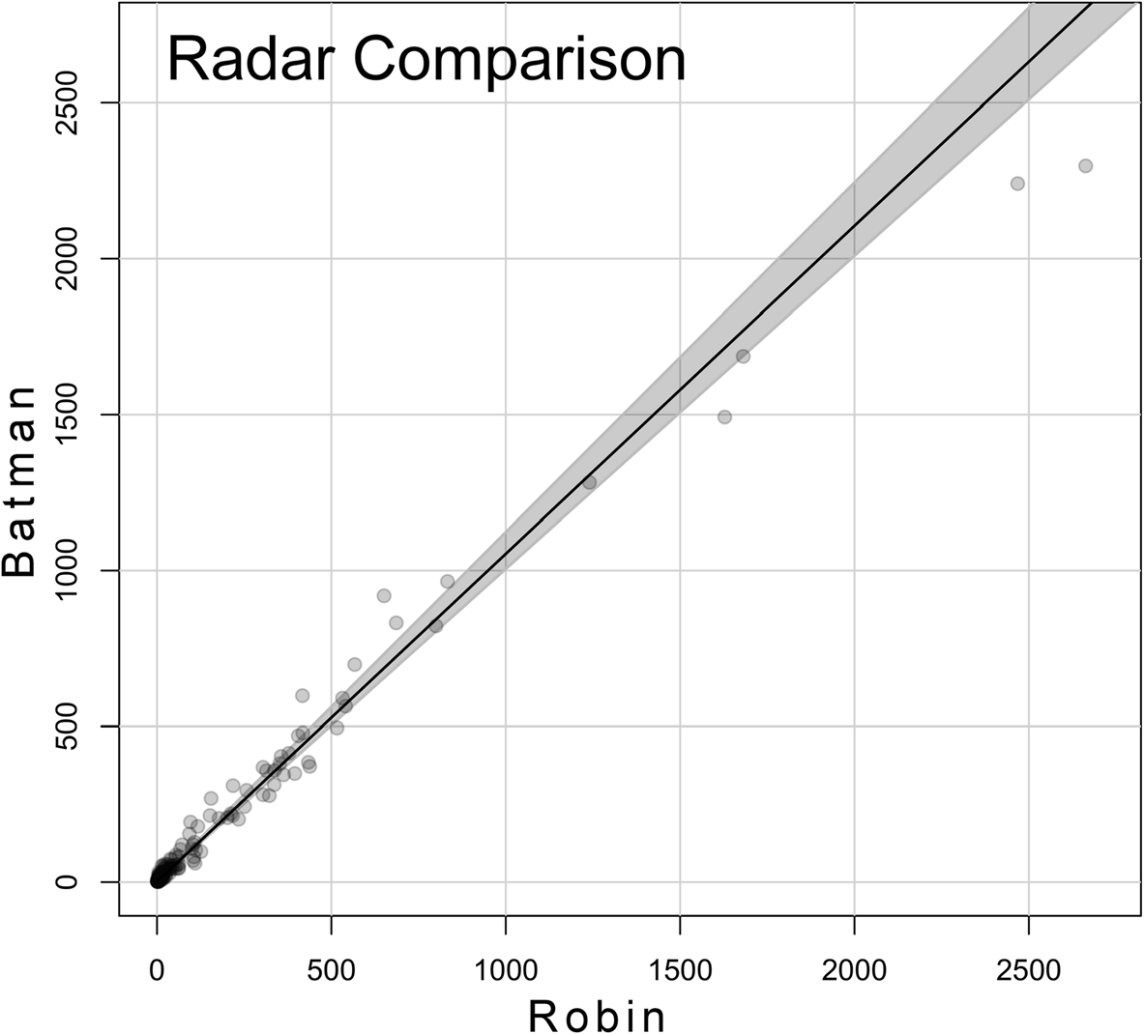
Slope test for the radar comparison. Each point represents the target passage rate (TPR) from Robin (x-axis) and Batman (y-axis) for each paired hour. Radar units were located in close proximity at two sites along the shoreline of Lake Michigan for the comparison. The line is a regression estimate of the relationship between passage rates recorded by the two units, and the shaded region is a bootstrapped 95% confidence interval for the regression line estimate

We recorded 145 h, including 85 nocturnal hours during which both radars were running simultaneously for the radar comparison. Hourly target counts during the radar comparison were highly correlated, indicating similar target detection and tracking rates between the units. The slope test produced a regression line slope not significantly different from 1:1 (slope estimate 1.05 with 95% CI 0.99, 1.11, with Batman TPR on the y-axis), indicating similar detection rates between the radar units. Target passage rates were slightly higher for Batman, but the difference was not large enough to indicate a systematic bias that would invalidate direct comparisons (Fig. 3). Assuming the radars were sampling the same bird and bat traffic due to their close proximity and parallel orientation, these results satisfied the requirement that detection rates were similar enough to compare target counts at shoreline and inland sites without adjusting for differences in radar performance.

### Migration activity

**Fig. 4:**
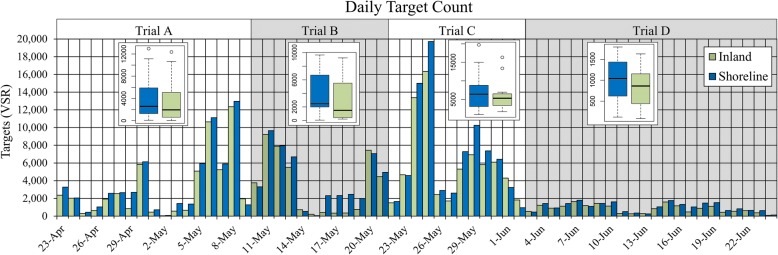
Time series of daily target counts collected on the vertical antenna (VSR) of each radar unit during the four trials. Each bar represents the total number of tracked targets collected during the 24-h period beginning and ending at midnight. Activity during the first three trials (A, B, and C) was sporadic with distinct peaks, typical of migration periods. Activity during trial D was reduced and consistent, thereby more indicative of post-migration breeding season activity and was omitted from further analysis. Inset boxplots summarize daily target counts within each trial, with a bold line representing the median and shaded boxes representing the interquartile range

Migration events, which are indicated by spikes in VSR target counts, were sporadic but occurred from the end of April (beginning of trial A) to the beginning of June (end of trial C). Migration events had largely subsided by the time we moved the radars to the final location for trial D at the beginning of June (Table 2, Fig. 4). Based on this distinct change in activity, we determined that the bulk of migration had ended at or near the beginning of trial D on June 2, and targets observed during trial D were less likely to be involved in migratory movements. Because the focus of this study was specifically on migrating birds and bats, trial D was omitted from formal analyses, specifically the regression slope tests and direction analysis.

**Table 2:** Target passage rate (TPR) summary statistics

	Paired Hours	TPR^a^ *r*	Pooled Mean TPR^b^ ± SE				Percent difference^c^ between shoreline and inland TPR ± SE			
Trial			Day	Dusk	Night	Dawn	Day	Dusk	Night	Dawn
RC	145	0.99	21 ± 5	30 ± 16	382 ± 103	24 ± 8	–	–	–	–
A	283	0.94	15 ± 6	14 ± 6	476 ± 84	58 ± 26	99 ± 69	211 ± 136	11 ± 8	203 ± 120
B	232	0.91	34 ± 8	46 ± 23	546 ± 108	164 ± 45	53 ± 18	13 ± 15	13 ± 12	143 ± 57
C	221	0.97	85 ± 14	56 ± 10	767 ± 137	189 ± 46	22 ± 18	26 ± 18	13 ± 6	124 ± 52
D	457	0.77	19 ± 2	18 ± 2	92 ± 7	21 ± 7	(10 ± 10)	11 ± 11	34 ± 9	(39 ± 43)

Number of paired data collection hours, Pearson’s correlation coefficient (*r*) for passage rate between radar units per paired hour, passage rate per biological period, and percent difference in passage rates between shoreline and inland sites per biological period for four trials and radar comparison (RC). ^a^ TPR is the number of targets detected by the vertical antenna within the 1 km standard front per hour. ^b^ Pooled mean is the average hourly target passage rate, pooled between both radar units. ^c^ Percent difference was calculated as shoreline TPR minus inland TPR, divided by inland TPR. Percent difference is reported as a proportion of inland TPR for comparability to slope test results. The mean difference with associated standard error is reported. A positive value indicates higher activity at the shoreline. Negative values are in parentheses

#### Slope test

**Fig. 5:**
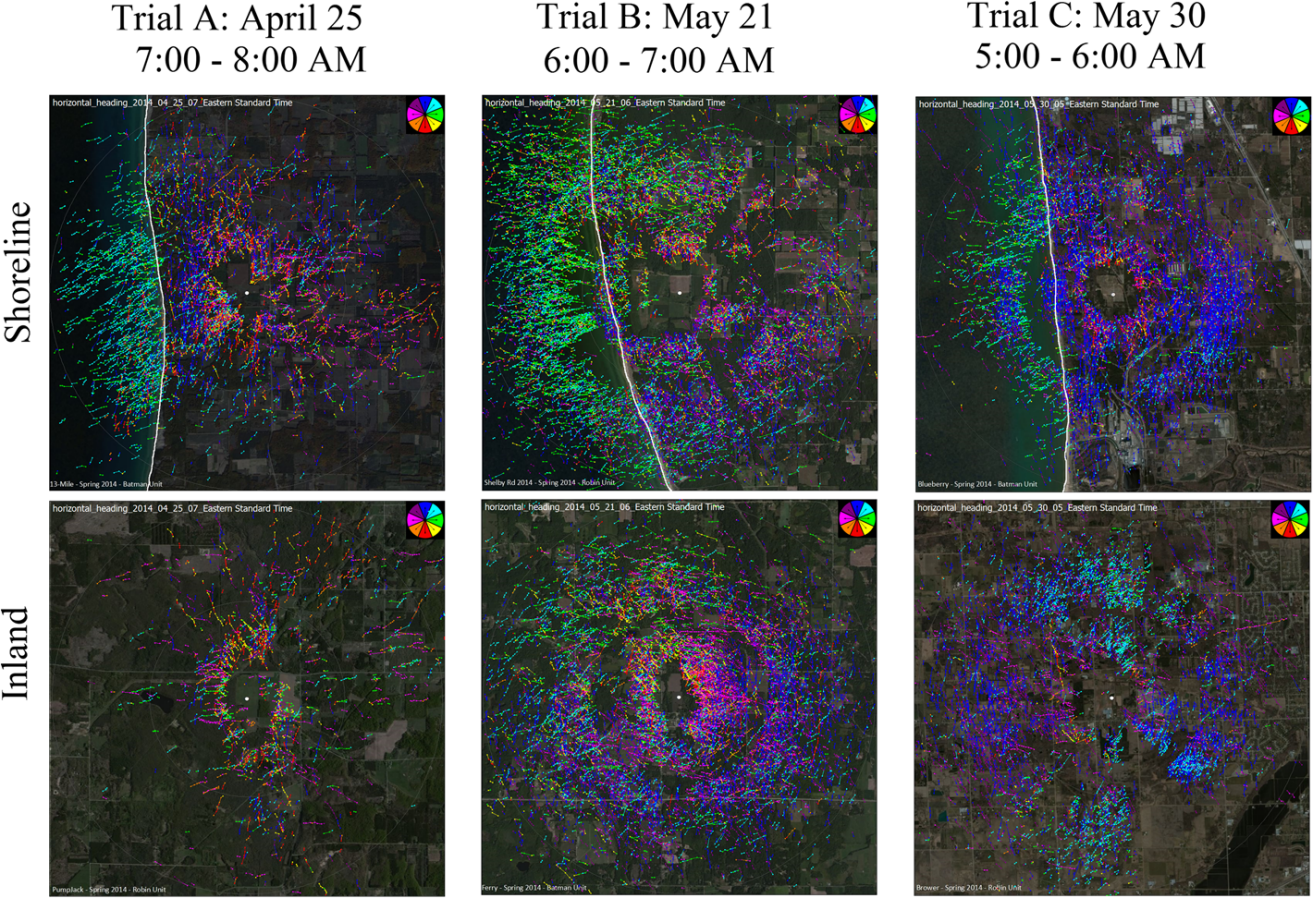
Example Trackplot images from the horizontal scanning radar that depict the direction of target movement during paired morning hours from shoreline and inland locations during trials A, B, and C. The color of each track indicates flight direction according to the color wheel in the upper right corner: dark blue for north, red for south, magenta for west, and green for east. The coastline is highlighted in white on the underlay of shoreline images. Lake Michigan is to the left of the white line. Targets returning to shore are light blue and green tracks on the left side of the shoreline images

**Fig. 6:**
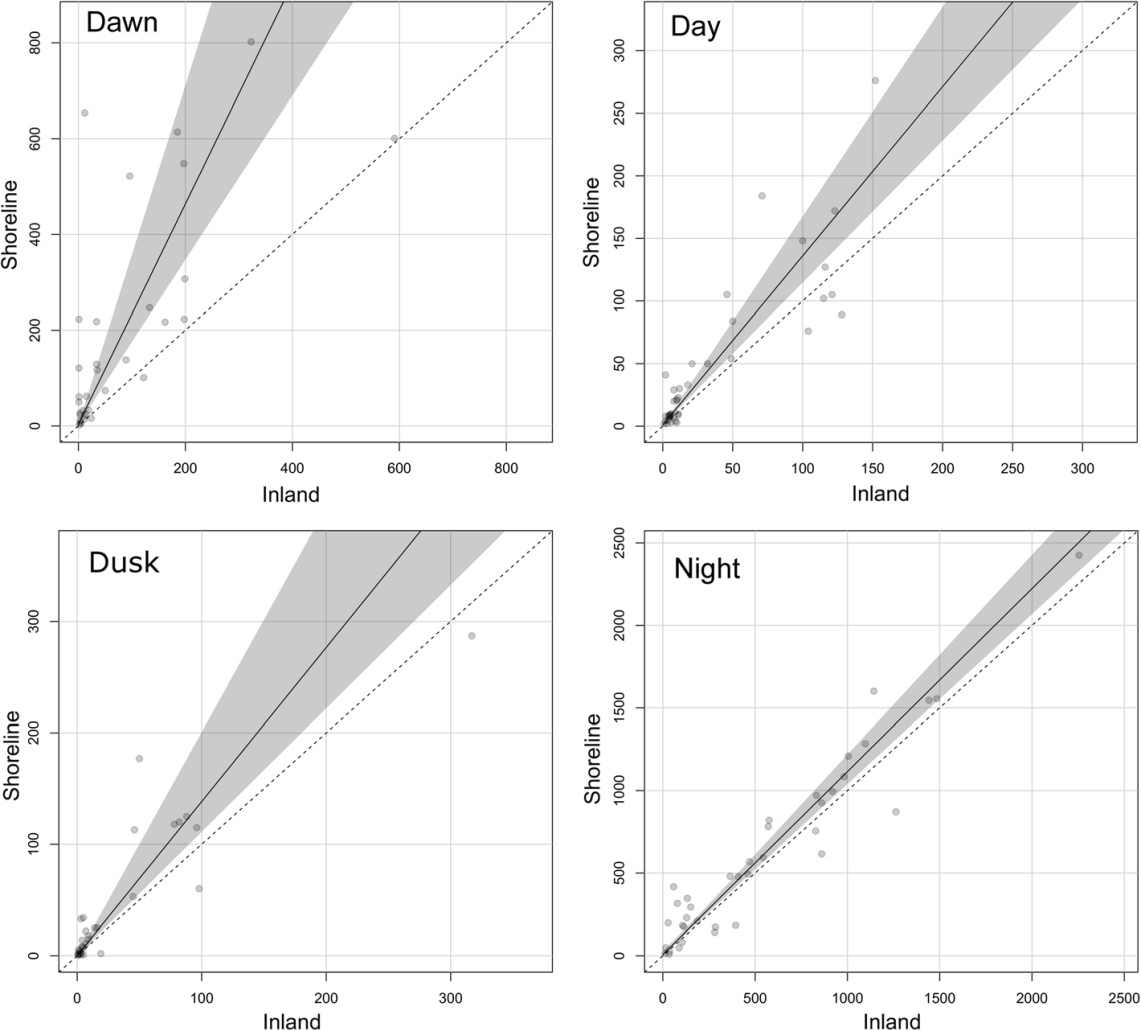
Comparison of dawn, day, dusk, and night target passage rate (TPR) at paired shoreline and inland sites. This analysis includes data from trials A, B, and C on the east coast of Lake Michigan during spring of 2014. The diagonal dotted line in each figure represents the 1:1 ratio that would be expected if passage rates were the same at shoreline and inland locations. The black line is the Poisson least rectangles regression line, representing the estimated relationship between shoreline and inland passage rates. The shaded confidence region spans the bootstrapped 95% confidence intervals of slope and intercept. Each point represents the mean TPR for a single biological period. Note the difference in axis scales among biological periods

We recorded more traffic at shoreline sites than at paired inland sites for each trial. During the migration season (trials A, B, and C), we estimated overall target passage rates to be 27% higher at shoreline sites than at inland sites (regression slope estimate = 1.27, Table 3). Differences in passage rates varied by biological time period, but shoreline activity was higher within each period. At night, when passage rates were highest, the slope test indicated a relatively small but significant difference of 11%, whereas the differential increased dramatically with the approach of sunrise. At dawn, we found a difference of 132%, meaning more than twice as many targets were observed at shoreline sites than at inland sites during that biological period (slope estimate 2.32, Table 3). The large difference at dawn is consistent with observations from the HSR that showed targets following the shoreline, and targets over water returning to shore just before and after sunrise (Fig. 5). During the day and at dusk, relatively low numbers of targets were detected, and these two periods had similar passage rate differences between shoreline and inland: 35% during the day and 37% at dusk. Confidence intervals around the regression estimates from the shoreline-inland comparison formed confidence regions entirely above the 1:1 line, indicating that shoreline passage rates were significantly higher than inland passage rates for each biological period (Fig. 6, Table 3).

**Table 3:** Regression estimates for shoreline-inland comparison

	All Periods	Day	Dusk	Night	Dawn
Slope	1.27	1.35	1.37	1.11	2.32
CI	1.15, 1.42	1.08, 1.65	1.04, 1.91	1.01, 1.21	1.63, 3.53
Intercept	0.96	1.13	0.13	6.39	1.98
CI	0.82, 0.99	−0.65, 2.39	−1.12, 0.84	−9.02, 22.96	1.36, 5.95

Estimated slope and intercept of Poisson least-rectangles regression comparing shoreline TPR (y-axis) to inland TPR (x-axis) for each biological period, as well as all periods combined, over trials A, B, and C. Slopes greater than 1 indicate higher activity at the shoreline. CI is the bootstrapped 95% confidence interval for the estimate

### Direction of flight

**Fig. 7:**
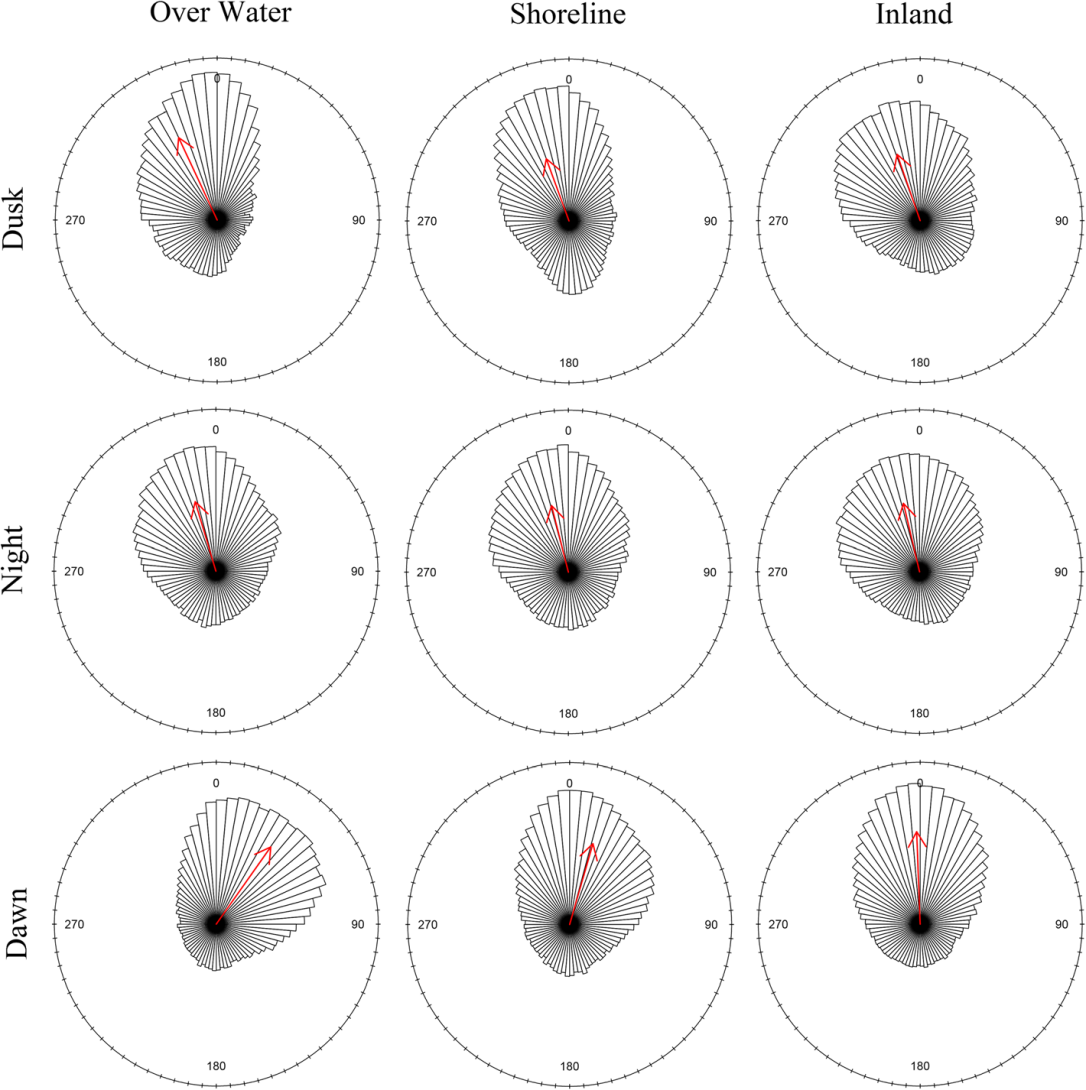
Distribution of targets by direction of flight. Zero degrees denotes north and each wedge represents 5 compass degrees. Each rose diagram represents 60,000 sample targets (20,000 from each trial A, B, and C). Over-water targets are a subset of all targets observed at the shoreline locations. Arrows point toward the circular mean target direction, and arrow length is the mean resultant length (MRL) with a full radius representing perfect alignment (MRL = 1). Diagrams were produced using R package ‘circular’ [40]

Targets generally flew in a northern direction most nights, consistent with spring migration (Fig. 7). During dusk and night, flights were frequently oriented toward the northwest as well. Dusk flights at the shoreline and inland had nearly the same mean direction (340° and 341° respectively), but inland sites appeared to have more targets flying westward and fewer targets flying southward. At night, the difference in westward traffic largely disappeared, suggesting that large numbers of migrants were embarking on lake-crossing flights. At dawn, directional patterns shifted substantially. While inland flights oriented to the north, targets at shoreline sites redirected eastward, diverting from the prevailing northwest heading of nocturnal migration. The difference was most pronounced at the shoreline sites, and especially among targets flying over water, consistent with migrants returning to land.

Mean resultant length, a measure of directional concentration on a scale of 0 (uniform, or lack of directionality) to 1 (perfect alignment), was moderate among all sets of targets, ranging from 0.42 for shoreline targets at night to 0.59 for targets over water at dawn. Directionality was less concentrated at the inland sites generally. Flights at the shoreline, including over-water flights, appeared to be more affected by the presence of the coast, with flights aligned in a north-south orientation during dusk and night, and then turning east at dawn. Directional concentration can result from both large numbers of targets flying in a similar direction and a lack of targets flying in the opposite direction. For example, targets over water at dusk seldom flew to the east or southeast, resulting in a relatively high concentration (0.56) to the northwest (335°), even though the largest numbers of targets were flying due north.

We tested the dawn reorientation hypothesis with Watson-Williams tests comparing mean direction during the night to mean direction at dawn. The change in directionality from night to dawn was positive (clockwise) at both shoreline and inland sites. The size of this shift to the east varied among trials at inland sites, but was consistent and large for the shoreline sites. The Watson-Williams tests indicated a significant directional shift for all comparison groups except the inland site during trial A, where mean direction at night and dawn were nearly identical (Table 4). Low *p*-values for groups with directional shifts are partly attributable to large sample size and do not necessarily indicate biological significance. However, the magnitude of dawn’s effect, measured as degrees difference between mean directions, does reflect a change in flight behavior that is biologically meaningful. Whereas inland targets did have a significant shift of 11 degrees over all trials, the shoreline sites averaged a much larger shift of 30 degrees, and those over water exhibited a 52 degree shift from north-northwest (343°) to northeast (35°). The large-scale reorientation of targets indicated a strong attraction effect of the coastline occurring around sunrise, and that the effect was most intense for migrants flying over water.

**Table 4:** Results of Watson-Williams tests for difference in mean direction between night and dawn

Location	Trial(s)	df1	df2^a^	*F* statistic	*p*-value	Difference ^b^ (degrees)
Inland	All trials	1	119,998	740	≤ 0.001	11.11
	Trial A	1	39,998	0	0.963	0.03
	Trial B	1	39,998	729	≤ 0.001	22.14
	Trial C	1	39,998	144	≤ 0.001	6.98
Shoreline	All trials	1	119,998	5048	≤ 0.001	30.34
	Trial A	1	39,998	1756	≤ 0.001	31.69
	Trial B	1	39,998	1772	≤ 0.001	35.13
	Trial C	1	39,998	1370	≤ 0.001	24.09
Over Water	All trials	1	119,998	17,098	≤ 0.001	51.84
	Trial A	1	39,998	7153	≤ 0.001	57.67
	Trial B	1	39,998	5292	≤ 0.001	58.74
	Trial C	1	39,998	4944	≤ 0.001	41.84

^a^A random sample of 20,000 targets per biological period were included in each trial-specific test. Samples were pooled for the all-trial tests (*n* = 120,000). Tests were conducted using R package ‘circular’ [40]. ^b^ Difference is the change in mean flight direction between night and dawn. Positive values indicate a clockwise shift

## Discussion

We provide evidence that aerial migrant activity is higher near the shoreline compared to inland locations. Using methods that addressed observational bias and accounted for extraneous spatial and temporal variation in migrant activity gave us confidence that the patterns we observed are biologically meaningful and broadly applicable. Radar studies are inevitably affected by the location of the radar itself (by clutter, increasing sample volume with distance from the radar, and distance-dependent detection rates), but by systematically relocating our radar units we were able to address both radar-specific and site-specific effects on detection rates. First, by co-locating the units for several nights at the beginning of the season we ensured that results would be comparable between units. Second, by moving the units among multiple trial locations and alternating units between shoreline and inland treatments, we were able to demonstrate that differences in observed activity were representative of large-scale migration patterns along the coast of Lake Michigan, rather than a product of local habitat features. Further, continuous and concurrent monitoring for at least ten days at each pair of trial sites reduced the potential for daily and seasonal variation in migrant passage rates to obscure overall migratory trends.

Radar can be a powerful tool, but it should be used with careful consideration of its limitations. The principal limitation of radar technology is the lack of taxonomic specificity provided. All flying animals are classified as “targets.” The use of S-band frequencies reduces, but does not eliminate, interference from precipitation and unintentional detection of insects. All returns identified by the tracking system as targets were considered to be flying vertebrates, but may have included invertebrates or other airborne objects. Birds, and especially nocturnal migrant passerines, could not be differentiated from bats. Additionally, the sample volume is an irregular shape, which obscures much of the airspace near the radar unit, and causes double-counting, especially on the horizontal antenna. Ground clutter and unpredictable radar artifacts further reduce detection rates within particular areas of the sample volume. Even with these limitations, we are confident that the data provided here are a valid representation of passage rates and flight behaviors of migrants using the study airspace. Given proper study design, careful site selection, and an awareness of each unit’s shortcomings, radar can gather information on nocturnal migrants that no other method can.

Our paired radar units provided relatively small windows into a massive event in which millions of nocturnal migrants cross over the landscape following similar environmental cues and direction [14, 41]. This broad-front migration has been well documented at weather stations across much of the US [42]. Our observations were largely consistent with previous findings in that the timing, relative intensity, and direction of nocturnal pulses recorded by our radars typically mirrored each other when looking at the broad pattern of movement. Despite similar patterns of nocturnal migration pulses throughout the season, we found clear differences in the absolute intensity of migration, and direction of flight at dawn between shoreline and inland sites. Consistent with radar observations from the Gulf of Mexico [22], we found that migrant densities were highest near the shoreline and significantly lower inland. This effect of the shoreline on migrants, while varying in magnitude, was evident during all biological time periods along the coast of Lake Michigan.

Differences between shoreline and inland activity occurred consistently throughout the migration season at sites that spanned over 150 km of coastline. The vast majority of flight activity we observed occurred at night, especially during the heavy migration period from late April until early June. As a result, the modest-sounding 11% difference between shoreline and inland activity at night represents a large number of migrants. Shoreline activity was clearly higher than inland activity at night, but the lake did not appear to present a significant deterrent during those hours. We regularly observed migrants flying to the west and northwest from the shoreline at night, likely embarking on lake-crossing flights. Migrants *en route*, flying at altitude, will stay aloft for the course of the night and can cross the lake in about 2.5 h, assuming a 12 m/s flight speed [43]. Migrants embarking on long-distance water crossings have also been documented along ecological barriers that would take considerably more time to cross, such as the Gulf of Mexico [44], northern Atlantic Ocean [45], and Mediterranean Sea [26, 46]. These observations reinforce the concept of an ecological barrier as a deterrent to landing but not necessarily to crossing flights. Nonetheless, during this time period when we expect the shoreline effect to be the weakest, we still found significantly more traffic above the shoreline sites.

Along the shoreline we studied, the strongest influence of the lake appeared near sunrise, as migrants turned back toward land. Presumably these were nocturnal migrant birds and bats seeking land for fuel or refuge as daylight approached [47, 48] and this behavior likely contributed to the largest proportional differences in target passage that we observed. At dawn, shoreline passage rates increased to 2.32 times as high as passage rates inland. This increase in activity near the shore occurred consistently at dawn before dropping off during the day. Daytime airspace use was low, suggesting migrants were using the shoreline either for rest and refueling, or for immediate access to safe (terrestrial) landing spots before relocating inland via low-altitude flights. High nighttime and dawn traffic indicates that many birds and bats encounter terrestrial habitats along the shoreline, but the amount of time migrants spend in near-shore habitat may depend on weather conditions surrounding their arrival and eventual departure [49]. The non-specific nature of radar observation prevents us from linking dawn arrivals to dusk departures, but the length of stopover and the types of resources needed by migrants are likely to vary widely, depending on a combination of environmental factors and the condition of the individual migrant [50]. The consistency of dawn arrivals among trials indicates that the entire shoreline serves as stopover habitat in one capacity or another.

We began to observe influences of the lake on migrant activity again at dusk when flights at the shoreline appear to align in a more north-south orientation than at inland locations. However, the proportional difference in target passage at dusk was considerably less than that at dawn. High shoreline activity at dawn (when nocturnal migrants are arriving), combined with low and more uniform activity at dusk (when nocturnal migrants are departing) suggests high stopover use at the shoreline, but also indicates stopover use across the entire study area that varies temporally and spatially. We propose two factors that may contribute to the observed differences between dawn and dusk: 1) initiation of nocturnal flights, the timing of which can be highly variable [18, 51, 52], occurred over a broader period of time than the abrupt termination of flights at dawn, or 2) many migrants terminating flights at the shoreline relocated more than 20 km inland prior to subsequent migratory flights [47, 53, 54]. The fact that overall activity was lower during dusk (Table 2) supports the first explanation: assuming that over the course of the season the number of migrants arriving at the study area equals the number of migrants departing the study area, the traffic rate at dusk was not adequate to offset dawn arrivals. Targets departing later in the evening were classified as night flights according to our biological period definitions. Migrants departing early in the night from longitudes in between the two radar units could partially account for the 11% higher passage rate near the shoreline (although the nighttime differential can also be explained by migrants moving north along the coast). However, the slope test finding that passage rates were far more even between sites at dusk than at dawn suggests substantial post-migration dispersion did occur. Inland dispersion can be particularly intense at coastlines after large migration nights and nights when winds push migrants off course [55]. These “morning flights” may account for some of the dawn activity at the shoreline, and would also explain the more even distribution of birds entering the airspace at dusk. A lull in activity during the day was a consistent feature at this shoreline as well as other Great Lakes sites (e.g. [30]). The lack of daytime bird activity on radar suggests that morning flights either occur soon after sunrise and do not continue into the day, or are limited to relatively low altitudes (below 100 m), where detection by radar is limited, consistent with a desire to avoid predation.

Many factors likely play a role in determining migration routes of birds, including ecological barrier effects, food abundance, habitat preferences for stopover or long-distance flight staging, weather patterns, and climate trends [2, 8]. Additionally, each of these factors may affect guilds or individual species differently. Far less is known about the effect these factors may have on the migratory pathways of bats. Our observations of aerial activity support a broad and simple mechanistic explanation of one factor contributing to shoreline accumulation that is by no means new [24, 27]: nocturnal migrants flying over water return to land at dawn. Meanwhile, migrants flying over land continue (for the time being) in the predominant migratory direction, and this results in a confluence of migrants along the shoreline.

The turn to shore itself appears to represent some degree of inefficiency in the migration strategy of nighttime flyers. Dispersing over water or embarking on lake-crossing flights from the shoreline late in the night, only to return to shore at dawn, causes an unnecessary expenditure of time and energy. Changes to flight direction add up to longer overall journeys and the use of more valuable physiological resources during this sensitive life stage [1, 56]. Longer journeys could also delay arrival at breeding grounds and lower the probability of reproductive success [57]. However, given the relatively short distance across Lake Michigan (on the scale of trans-continental migration), the loss of travel time and fat reserves incurred by dawn reorientation might be negligible [58]. Even if migrants are aware of the transition from land to water, the tendency to maintain a direct course offers advantages. The potential benefits of reaching the other shoreline, which is closer to breeding grounds and less densely occupied [27], may outweigh the risks of being caught over water at daybreak.

Regardless of behavioral explanations, airspace along the shoreline of Lake Michigan was used more heavily than inland airspace during the migration season. We found higher shoreline passage at each pair of study sites along Lake Michigan, suggesting that these findings may be applicable to Great Lakes coasts more broadly, and perhaps informative for coastal areas in general. In theory, the magnitude of shoreline accumulation at dawn may be proportional to the number of terrestrial migrants flying over adjacent waterbodies during the night, potentially resulting in higher rates of accumulation for larger water bodies. Further research would be needed to confirm this hypothesis. However, the turn to shore is not the only cause of high migrant use of shorelines. Factors such as stopover habitat quality and the use of coasts as guiding lines likely contribute as well [12, 20]. Considering all these factors together may provide a more accurate explanation of why we observe more migrants along the coast of Lake Michigan and elsewhere.

## Conclusions

This study provides direct evidence that shorelines are areas of especially high use by migrants, and evidence of a dawn turn toward shore that contributes to the differences in activity rates we observed. Migrants use coastlines more frequently than inland areas for purposes of stopover and refuge, if only for the fact that more migrants terminate flights along shorelines to avoid flying over water during the day. These results suggest that the Great Lakes affect nocturnal migrants in a manner similar to large ecological barriers [24, 46], and our findings of a dawn turn towards shore suggest migrants may need to cope with these spans of unhospitable environment unexpectedly. Additional work investigating how large water bodies affect migration, especially decision-making by migrants starting their journeys (potentially hundreds of kilometers from the shore), is still needed. In addition, conservation of habitat in shoreline areas, with an emphasis on providing resources for migrating birds and bats, may benefit more migrants per hectare than other areas, simply due to higher rates of use. Anthropogenic activities that put birds and bats at risk should recognize that shoreline concentration of migrants, which may not be fully evident from diurnal or intermittent surveys, indicates that impacts from development, land use, or habitat alteration may cause additional harm if occurring near shorelines.

## Additional files

Additional file 1: VSR activity data. Radar data from vertical scanning radar (VSR) antenna used to measure target passage rate (TPR). (XLSX 106 kb)

Additional file 2: Slope test script. R script for Poisson least rectangles regression slope test used in radar comparison and shoreline-to-inland comparisons. (R 4 kb)

Additional file 3: Batman direction data. Radar data from horizontal surveillance radar (HSR) antenna on Batman unit, used in analyses of flight direction. (XLSX 12941 kb)

Additional file 4: Robin direction data. Radar data from horizontal surveillance radar (HSR) antenna on Robin unit, used in analyses of flight direction. (XLSX 10359 kb)

## Abbreviations

df: degrees of freedom
HSR: horizontal surveillance radar
RC: radar comparison
SE: standard error
TPR: target passage rate
VSR: vertical scanning radar
